# Serum calcium level is related to both intima-media thickness and carotid atherosclerosis: a neglect risk factor in obese/overweight subjects

**DOI:** 10.1186/1479-5876-10-114

**Published:** 2012-06-06

**Authors:** Tiziana Montalcini, Gaetano Gorgone, Arturo Pujia

**Affiliations:** 1 Clinical Nutrition Unit, Department of Medical and Surgical Science, School of Medicine, University Magna Grecia, Viale S Venuta, 88100 Catanzaro, Italy

**Keywords:** Calcium, Atherosclerosis, Carotid, Obesity, Ultrasound

## Abstract

**Background:**

Experimental studies suggested that high serum calcium may be important in the pathogenesis of vascular diseases. Since calcium seems to affect specifically the cerebrovascular district, aim of this study was to determine the relation between serum calcium levels, within normal range, and subclinical atherosclerosis in the carotid arteries, in a population of obese/overweight subjects.

**Methods:**

In our retrospective study we included 472 subjects (59% female) with body mass index equal to or more than 25 kg/m^2^. They underwent a physical examination, a biochemical assessment (including calcium evaluation) and a B-mode ultrasonography of the extracranial carotid arteries to detect carotid atherosclerosis presence and to measure intima-media thickness.

**Results:**

Mean age of the population was 50 ±12 years. Prevalence of the Carotid atherosclerosis was 40%. Mean carotid intima-media thickness was 0,66 ± 0,18 mm. The univariate and multivariate analysis showed an association between calcium and carotid intima-media thickness (p = 0,035). We divided the population in serum calcium tertiles. We found an higher carotid atherosclerosis prevalence in the III tertile in comparison to that of the I tertile (p = 0,039).

**Conclusions:**

In this study we found a positive relation between serum calcium levels, within normal range, and subclinical atherosclerosis in the carotid arteries, in a population of obese/overweight subjects. It is important to consider the impact of the serum calcium levels in the overall risk assessment of patients, at least in obese subjects.

## Background

Experimental studies suggested that calcium-phosphate metabolism could influence the pathogenesis of vascular diseases. In fact, several studies showed an increased cardiovascular morbidity and mortality in primary hyperparathyroidism (PHPT)[1-8]. Furthermore, increased carotid intima-media thickness (CIMT), a subclinical predictor of coronary and cerebrovascular events [9-12], was found in patients with PHPT and high calcium levels [13], suggesting a causative role of hypercalcemia. However, in most patients with PHPT, serum calcium levels is in normal range [9]. A study showed that serum calcium level, within the normal range, was positively associated to carotid atherosclerotic plaques presence [14]. Consequently, to date, the link between the serum calcium level and the cardiovascular diseases has not been fully elucidated. Furthermore, calcium and phosphate levels seem to exert differential effects, depending on the type and location of the vascular bed [15]. In particular it was demonstrated that calcium seems to affect specifically the cerebrovascular district [15]. For this reason our objective was to investigate on the possible relation between serum calcium levels, within normal range, and the presence of carotid atherosclerosis. Furthermore, since a recent research [16] proposing the participation of the calcium-sensing receptor (CaSR) as a possible link between obesity and inflammation, had attracted our attention, we chose to investigate the relation between calcium and carotid atherosclerosis in a population of overweight/obese subjects.

## Methods

In our retrospective study participants were recruited from subjects underwent an health-screening tests in our hospital clinic for the presence of one or more cardiovascular risk factors (obesity, hyperlipidemia, hypertension, diabetes, smoking).

A total of 640 individuals, with at least one cardiovascular risk factor, underwent an ultrasonography of the carotid arteries during a period from the year 2008 to 2011. We excluded 168 normal weight individuals (40% males), therefore in this study 472 subjects (59% women; 41% men) were included. All individuals were Caucasian and underwent a collection of the medical history, by a standardized questionnaire administered to obtain information about current and past medication use, smoking habits and presence of cardiovascular disease. They underwent, also, a physical examination including the evaluation of the body mass index (BMI)(calculated as weight, in kg, divided by square of height, in meter); the measurement of the waist circumferences (WC)(a measuring tape was used for WC, measured right below the ribs); the measurement of the systemic blood pressure in both arms by a sphygmomanometer (systolic and diastolic blood pressure - SBP and DBP). The ankle-brachial systolic pressure index (Winsor Index) was determined by dividing the highest of the posterior tibial or dorsalis pedis systolic blood pressure by the highest brachial pressure. On the basis of the clinical history, no subjects had symptomatic cardiovascular disease.

Venous blood was collected after fasting overnight into vacutainer tubes (Becton & Dickinson) and centrifuged within 4 h. Serum glucose, creatinine, total cholesterol, high density lipoprotein cholesterol, triglycerides and calcium were measured with enzymatic colorimetric test (see Additional file 1: Supplemental materials). Serum Calcium were measured by Schwarzenbach test (see Additional file 1: Supplemental materials). Quality control was assessed daily for all determinations. The investigation conforms to the principles outlined in the Declaration of Helsinki. All patients provided informed consent to participate in our research and to use their data.

The following criteria were used to define the cardiovascular risk factors; diabetes: fasting blood glucose ≥126 mg/dl or antidiabetic treatment; hyperlipidemia: total cholesterol >200 mg/dl and/or triglycerides >200 mg/dl or lipid lowering drugs use; hypertension: systolic blood pressure ≥140 mmHg and/or diastolic blood pressure ≥90 mmHg or antihypertensive treatment; smoking: present smokers; overweight: BMI ≥ 25 < 30 Kg/m^2^, obesity: BMI ≥ 30 kg/m^2^.

### Ultrasound

The subjects underwent a B-mode ultrasonography of the extracranial carotid arteries by use of a duplex system (an high resolution ultrasound instrument “Advanced Technology Laboratories” -ATL, High Definition Imaging –HDI 5000 with a 5- to 12-MHz linear array multifrequency transducer). All the examinations were performed by the same ultrasonographer blinded to clinical information. All patients rested in the supine position for at least 10 min before the study and were kept in this position during the procedure. Electrocardiographic (ECG) leads were attached to the ultrasound recorder for on-line continuous heart rate monitoring. The right and left common (CCA) and internal carotid arteries (including bifurcations) were evaluated with the head of the subjects turned away from the sonographer and the neck extended with mild rotation. The CIMT, defined as the distance between the intimal–luminal interface and the medial–adventitial interface, was measured as previously described [17]. Briefly, in posterior approach and with the sound beam set perpendicular to the arterial surface, 1 cm from the bifurcation, three longitudinal measurements of IMT were completed on the right and left common carotid arteries far-wall, at sites free of any discrete plaques. The mean of the three right and left longitudinal measurements was then calculated. Then, we calculated and used for statistical analysis the mean CIMT between right and left CCA. Plaque, detected in longitudinal and transverse planes with anterior, lateral and posterior approaches, was defined as an echogenic focal structure encroaching into the vessel lumen of at least 50% of the surrounding CIMT value. Stenosis was defined as a peak systolic velocity >120 cm/s and occlusion was defined as absence of Doppler signal. According to these criteria, subjects were considered as normal if no lesion was detected, or having carotid atherosclerosis when a plaque, stenosis or occlusion was detected in at least one segment of common, bifurcation or internal carotid artery. The coefficient variation of the methods was 3.3%.

### Statistical analysis

Data are reported as mean ± S.D. The *χ*2-test was used to compare the prevalence among the groups. Univariate analysis was performed to identify the factors related to CIMT. The Multivariate regression analysis was performed to test for confounding variables and data were adjusted for gender. The Logistic regression analysis was used to test variables correlated to carotid atherosclerosis presence, including only the factors different at comparison (*t*-test) between subjects with and without carotid atherosclerosis. Furthermore, the *χ*2-test was performed to compare the prevalence of the carotid atherosclerosis between “calcium tertiles” of population (between I and III tertile). Significant differences were assumed to be present at *p* < 0.05. All comparisons were performed using the SPSS 17.0 for Windows 233 S. Wacker Drive, Chicago, Illinois 60606, USA.

## Results

**Figure 1:**
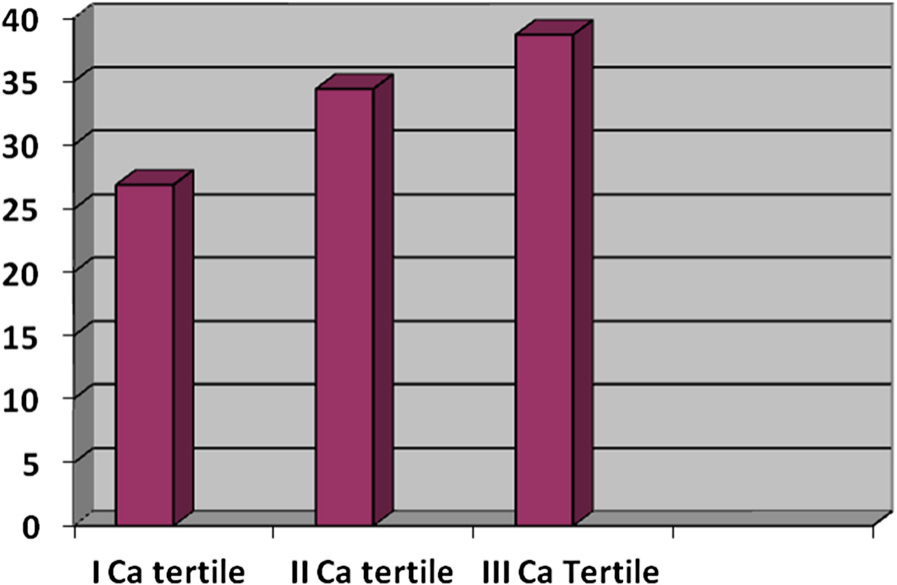
Carotid Atherosclerosis prevalence between calcium tertiles (p = 0,039).

The mean age of the population was 50 ± 12. The BMI was 33 ± 6. The characteristics of the overall population are showed in Table 1. Subjects treated with lipid lowering drugs were 28.6%. Table 2 shows the Multivariate regression analysis, adjusted for gender, including the variables correlated to CIMT at univariate analysis (age, glucose, SBP, DBP calcium, WC - data no showed). This analysis confirmed the association between calcium and CIMT, as well as, age, PAS, WC with CIMT (Table 2). In this population the prevalence of the Atherosclerotic was 40% (Table 1). After *t*-test between subjects with and without carotid atherosclerosis (Table 3), we performed a Logistic regression analysis with Carotid Atherosclerosis as dependent variable (Table 4). In this analysis, in the first two models performed, including age and gender (I model), and age, gender and WC (II model) calcium was correlated to Carotid Atherosclerosis. However, when (in the third model) glucose and SBP were included in the analysis, calcium was excluded (data no showed). Finally, we divided the population in serum calcium tertiles. Figure 1 depicts the higher carotid atherosclerosis prevalence of the III tertile in comparison to that of the I tertile (p = 0,039).

**Table 1:** Characteristics of the population

**Variable**	**Mean**	**SD**
Age (years)	50	12
BMI	33,3	5,9
Calcium (mg/dl)	9,4	0,4
Creatinine(mg/dl)	0,8	0,6
Glucose(mg/dl)	101	26
TCholesterol(mg/dl)	220	44
LDLcholesterol(mg/dl)	141	56
HDL(mg/dl)	50	14
Trygliceride(mg/dl)	174,46	106,9
CIMTmean (mm)	0,66	0,18
IWright	1,06	0,006
IWleft	1,07	0,007
Waist Circum(cm)	104,28	13,30
DBP(mmHg)	77,05	9,21
SBP (mmHg)	125,19	14,65
Carotid atherosclerosis (%)	40	
diabetes(%)	9,7	
Hyperlipidemia(%)	47	
hypertension(%)	25,8	
smokers(%)	7,4	
Female (%)	59	

**Table 2:** Multivariate regression analysis

**Dependent Variable: CIMT**	**SE**	**Beta**	**t**	**p**
Age	0,001	0,432	10,16	<0,001
SBP	0,001	0191	4,50	<0,001
WC	0,001	0,101	2,53	0,012
calcium	0,019	0,084	2,11	0,035

(the data are adjusted for gender)

Excluded variables: PAD, glucose (factors correlated to CIMT at univariate analysis-data no shown).

**Table 3:** **Comparison (*t*-test) between subjects with and without carotid atherosclerosis **

**Variables**	**Atherosclerosis presence**	**Atherosclerosis absence**	**p**
AGE (years)	57,42 ± 8	45 ± 11	<0,001
SBP(mmHg)	129 ± 15	122 ± 14	<0,001
DBP(mmHg)	77 ± 10	77 ± 9	0,334
WC (cm)	103 ± 13	106 ± 14	0,049
BMI	32 ± 5	34 ± 6	<0,001
Calcium (mg/dl)	9,4 ± 0,3	9,3 ± 0,4	0,029
Tot cholesterol (mg/dl)	222 ± 46	217 ± 43	0,116
HDL (mg/dl)	51 ± 14	50 ± 13	0,413

**Table 4:** Logistic regression analysis

**Dependent variable: Carotid Atherosclerosis**						
**I model**						
Variable	B	S.E.	Wald	Sig	R	Exp(B)
AGE	0,15	0,01	95,23	<0,001	0,38	1,16
CALCIUM	0,63	0,30	4,34	0,03	0,06	1,89
GENDER	1,19	0,26	21,00	<0,001	0,17	3,29
**II model**						
AGE	0,15	0,01	95,50	<0,001	0,38	1,16
CALCIUM	0,58	0,30	3,56	0,05	0,05	1,79
GENDER	1,28	0,26	23,11	<0,001	0,18	3,60
WC	−0,02	0,00	7,78	<0,001	−0,09	0,97

## Discussion

In this study we found a positive relation between serum calcium levels, within normal range, and CIMT. As expected,CIMT was, also, associated, to the classical risk factors like age, gender, glucose, SBP, DBP and WC. However, after adjustment for confounding factors in the multivariate analysis, the association between calcium and CIMT remained, as well as, for age, SBP and WC only (Table 2). Furthermore, in this study we showed an higher carotid atherosclerosis prevalence in the population group having high serum calcium level (III tertile)in comparison to group with low serum calcium level (I tertile) [Figure 1].

This study may have a relevant place among the studies already present in the literature since we evaluated a large number of subjects (472 individuals),with a BMI > 25 (overweight/obese) excluding subjects having pathological values of serum calcium. Furthermore, our study is important since investigated, at the same time, the relation between calcium and the various manifestations of the atherosclerotic process in the carotid district (i.e.: CIMT and plaques).

Similarly to other studies [12,15,18] the parathyroid hormone (PTH) measurement was not available. However, we had the objective to investigate on the possible association of serum calcium and subclinical atherosclerosis at carotid site, with others classical risk factors and not other factors.

Several investigations demonstrated the association between serum calcium and stroke, but not with coronary heart disease, in contrast to phosphate [15]. Although the cause of this divergence is uncertain, it was speculate that calcium-phosphate levels could exert differential effects, depending on the type and location of the vascular bed [15]. Thus, our finding is in line with the concept that the effects of the serum calcium are prominent for the cerebral district than for other sites [15].

The majority of cross-sectional studies indicated that elevated levels of established cardiovascular risk factors are associated with an increased CIMT [19-21] including obesity and the insulin resistance condition. Certainly, our study was not designed to explain the mechanisms underlying the association finding in this study, however, some hypothesis may be advanced.

Several observations showed that serum calcium level, in the normal range, affect the risk of having Metabolic Syndrome (MetS) and diabetes [22]. This association may suggests a first possible mechanism by which calcium affect the intima-media thickness in our study, confirmed also by our finding of the strong relation between WC, a factor linked to MetS, and CIMT. Moreover, in our study there was a positive relation between serum calcium and glucose (p = 0,022; r = 0,166 - data no showed).

Furthermore, recently, Cifuentes et al. [16] reported that obesity-associated proinflammatory cytokines increased the CaSR protein expression in primary human adipocytes as well as in LS14 human adipose cell line. CaSR is a seven transmembrane receptor which plays a central role in regulating calcium homeostasis. In particular the activation of the CaSR maintains serum calcium at normal levels [23]. CaSR is a multifunctional protein inhibiting basal lipolysis in adipocytes [23], regulating the smooth muscle cells of vessels [24], involved also in the ischemic neuronal death [25]. All these observations could support the link between serum calcium, within normal range, and the CIMT. Furthermore, it was showed that obesity, as well as, the metabolic syndrome condition are associated to high level of serum fetuin-A, a protein playing an important role in bone metabolism, metabolic disorders, and central nervous system disorders such as ischemic stroke and neurodegenerative diseases [26,27]. Actually it is unknown whether fetuin-A is an exacerbating or a protective factor in the cardiovascular system, however, it may represent the link between inflammation, calcium and atherosclerosis in obesity, needing further investigations.

Our study has several strengths and limitations. The study population was relatively large and the young population studied should be useful for the early identification of the subclinical carotid artery disease. Our study investigated, also, the various manifestations of the atherosclerotic process in the carotid district.

A limitation of our study, similarly to other studies [12,18], may be that PTH and vitamin D levels were not available, as well as, fetuin A measurement.

## Conclusions

We believe it is important to consider the impact of the serum calcium levels in the overall risk assessment of patients, at least in obese individuals. Despite a number of studies on this issue, actually the blood calcium level is considered a neglect risk factor for vasculature, thus, we think is important to perform other studies on this factor. It is unclear whether this finding will have a therapeutic implication. For now, it could clarify some morphological changes of specific vascular districts.

## Abbreviation

PHPT: Primary hyperparathyroidism; CIMT: Carotid intima-media thickness; CaSR: Calcium-sensing receptor; BMI: Body mass index; WC: Waist circumferences; SBP: Systolic blood pressure; DBP: Diastolic blood pressure.

## Competing interest

There is no conflict of interest that could be perceived as prejudicing the impartiality of the research reported.

## Authors’ contributions

All authors participated sufficiently in the work to take public responsibility for its content. All authors read and approved the final manuscript.

## Funding

This research did not receive any specific grant from any funding agency in the public, commercial or not-for-profit sector.

## Supplementary Material

Additional file 1 Supplemental materials. Click here for file
