# Supplementary material for: Serum calcium level is related to both intima-media thickness and carotid atherosclerosis: a neglect risk factor in obese/overweight subjects
Source: J Transl Med. 2012 Jun 6;10:114. doi: 10.1186/1479-5876-10-114 (PMC3551795; doi:10.1186/1479-5876-10-114)
Supplement: Additional file 1 — Supplemental materials. [file 1479-5876-10-114-S1.docx]

Dear Editor,

We are very grateful to the reviewers; we shared all the consideration and appreciated all the suggestions; as a consequence the manuscript was corrected and the suggestions were enclosed in the manuscript.

Next we illustrated the point to point answers to the reviewers.

Tiziana Montalcini on behalf of the authors.

Reviewer 1

1. The measurement method is disputable. Why did the investigators only perform 3 measurements on the left and right common carotid artery? This is uncommon. The reader would expect more measurements (at least 10 measurements per arterial site).

A.: We agree with this comment whether follow-up studies on CIMT progression/regression are presented. In these cases, in fact, we need to measure micron differences,therefore the precision need is very high. In epidemiological studies, instead, three measures on each side are usually sufficient. This was well established by consensus statement published in 2008 (see following reference). Indeed, eventual bias in this measurement should be considered as a random bias present in the entire population, therefore they are not influencing the final results.

Reference: James H. Stein, et al. Use of Carotid Ultrasound to Identify Subclinical Vascular Disease and Evaluate Cardiovascular Disease Risk: A Consensus Statement from the American Society of Echocardiography Carotid Intima-Media Thickness Task Force. *Endorsed by the Society for Vascular Medicine* Journal of the American Society of Echocardiography February 2008, 21:93-111

2. Further, carotid plaque evaluation is maximal poor. The authors only write about plaque prevalence. What`s about 1) plaque-prevalence in respective segments (internal / external / common carotid artery, carotid bifurcation), 2) further characterization of plaques (for example: echogenicity), 3) classification of stenosis?

A.: This is a correct comment depending on a misuse of the word “plaque”. We apologize for this inconvenient; the correct term to be used is “carotid atherosclerosis” that, as explained in the section method, includes all the atherosclerotic lesions present in carotid arteries. Our purpose is to distinguish between “normal” and “atherosclerosis affected” carotid arteries. This approach was usually well accepted, as demonstrated by several published manuscript (see following):

-T Montalcini, et al Relation of Low Bone Mineral Density and Carotid Atherosclerosis in Postmenopausal Women. Am J Cardiol 2004 94:266-269

-T. Montalcini, et al. Association of LDL cholesterol with carotid atherosclerosis in menopausal women affected by metabolic syndrome.Nutr Metabolism Cardiovasc dis2005;15:368-72.

-T Montalcini et al . Carotid atherosclerosis associated to metabolic syndrome but not BMI in healthy menopausal women. Diabetes Res ClinPract. 2007; 76:378-82

- T. Montalcini, et al., Role of endogenous androgens on carotid atherosclerosis in non obese postmenopausal women. NutrMetabCardiovasc Dis 2007;17:705-11

- T. Montalcini, et al. Large brachial and common carotid artery diameter in postmenopausal women with carotid atherosclerosis.Atherosclerosis 2008; 196:443-8

3. The investigators only present data for obese subjects and they conclude that their results could have an impact for general patients? The reader misses data for “normal” subjects (normal weight).

A.: We changed this sentence and stated that these results may have an impact only in overweight/obese subjects.

4. The actual format of the manuscript is poor. It doesn`t give more insight or knowledge in the theme. The reader is a little bit confused about the insufficient data-presentation.

A.:We added other sentences to ameliorate this aspect.

5. I would suggest a complete revision of the manuscript. Please take care of the English language. There are some text passages that are incomprehensible.

A. :We corrected English language and we edit all the manuscript.

6. Special Comment.

A.: We also accepted all the special comments, modifying the phrases according to reviewer suggestions.

Abstract:

L 01: Which abnormalities? High or low values?

A.:We stated to which abnormalities we referred.

L 25: only subclinical atherosclerosis (not “the”)

A.: We deleted the article.

L 27: including 472 subjects: please divide in men and women (or % men)

A.:We divided in men and women

L 28: I don`t know biochemical parameter parameter – please explain

A.: We corrected this term.

L 30: only mean-age – not the mean age

A.: We deleted the article.

L 30: you need units (49.99 years)

A.: We added this.

L 30: Did you ever see: 49.99 years? Please use one character

A.: We corrected this.

L 30: Not the prevalence – only prevalence

A.:We corrected this.

L 30: atherosclerotic

A.: We modified this.

L 31: Not the linear regression – only linear regression

A.: We changed this.

L 32: Why did you only provide a p-value? The reader is interested in CIMT-values.

A.: We included CIMT values

\# Please give information about mean-CIMT # The abstract needs further information about the measurement method # What`s about gender differences? # Please give information about threshold values: obese – not obese

A.: We added all these data.

Introduction: L 52-L55: This is copy – paste from the abstract, isn`t it?

A.: We changed this part of manuscript.

L 58: This is a retrospective study. – This sentence is horrible!!! You can introduce with: In our retrospective study …

A.: We modified this sentence.

L 61: 640 individuals – gender prevalences?

A.: We added these data.

L 62: Please don`t begin your sentence with numbers, like “472 of them ...”

A.: We modified this phrase.

L 62: We need further information about the gender prevalence

A.:We added this date.

L 71: How did you measure the blood pressure? Manual measurements? Please explain.

A.: We included this information.

L 86: Why did you only include smoker (not also ex-smoker?)

A.: We usually consider ex smoker similar to never smoker because it is well known that smoking cessation is associated with a considerable and rapid benefit in decreasing the risk of stroke and cardiovascular disease, as demonstrated by these studies:

1. Kawachi I, et al C..Smoking cessation and time course of decreased risks of coronary heart disease in middle-aged women. Arch Intern Med. 1994; 154: 169–175.
2. Kawachi I, et al. Smoking cessation and decreased risk of stroke in women. JAMA. 1993; 269: 232–236.
3. Wannamethee SG, et al. Smoking cessation and the risk of stroke in middle-aged men. JAMA. 1995; 274: 155–160.

L 129: PAS and PAD: please explain the abbreviations for the first time

A.: We spelled out the acronyms.

Discussion

L 149: In line you defined “obese” as BMI #30 kg/m2

A. : In the method section we included the reference range for overweight definition. In fact our study includes not only obese subjects, but also overweight. Consequently in line 149 we considered subjects with BMI >25

L 154: I don`t understand the sentence: “... was not considered for this study”

A.: We corrected this point.

L 186: A limitation – not limitations

A.: We corrected this.

Table 1:Age, BMI, calcium, glucose, total cholesterol, LDL/HDL, triglycerides, ...: Please use only one character (for glucose, age, ... you don`t need a decimal point)

A.: We corrected these.

Presented figures 1 a/b are very poor. Is there any information for the y-axis? What does it mean: ats (yes/no)? I cannot understand the white (or yellow) small box. Where is the information I vs. III? I don`t see it in the present figure.

A.: We completely revised the figure.

**Reviewer 2**

This is an interesting study where the authors aimed to determine the relationship between serum calcium levels and the subclinical atherosclerosis in the carotid arteries. However, there were some problems which would influence the quality of this study.

1. In the part of introduction, authors should give a reason why they only chose obese patients.

A.: In the introduction we added a sentence to explain our choice

2. As we had known, that primary hyperparathyroidism might influence the serum calcium level and carotid arteriosclerosis, so, maybe it is important to show the data of patients’ PTH, although it was difficult for a retrospective study to collect these data.

A.: We are in agreement with this comment but we did not measure it, therefore we cannot provide the PTH for all population. However, it is well known that also in the ARIC study parathyroid hormone and vitamin D levels were not available. Authors explained that “the potential influence of these hormonal axes on their study findings is a matter of conjecture”. We also stated that our study cannot be generalized to other populations and it could represent a stimulus to perform further prospective research in this issue.

3. Have the full name for ECG, ALT, etc, when these abbreviations appeared in paper at the first time.

A.: We added this.

4. The methods' description is not sufficient. Authors should provide methods that they used to examine serum glucose, creatinine, total cholesterol, high density lipoprotein cholesterol, triglycerides and calcium. If their description is presented elsewhere, please provide appropriate references.

A.: We included the method and reference as supplemental materials, in an “additional file”.

5. I suggest making correlation analysis before making Logistic regression analysis, and adding several scatter plots to show the relationship between age, gender, calcium, WC and carotid IMT.

A. Since carotid atherosclerosis is a dichotomous variable we preferred to make a t-test and to select the variables resulted statistically different. We added only a table with this t-test; indeed we think that the manuscript would result redundant in the case added 4 more figures to show a simple relationship between variables.

6. In the table 1, authors should provide normal range of serum glucose, creatinine, total cholesterol, high density lipoprotein cholesterol, triglycerides and calcium.

A. This is an unusual request, we never see normal range on published manuscript even in manuscript accepted by this journal(see Tarantino et al *Journal of Translational Medicine* 2012, 10:50). Indeed we specified in method section all the criteria used to define the cardiovascular risk factors based on the above-indicated parameters.

7. Authors chose obese/overweight patients to make the study. The mean serum TCholesterol level is 220.19 mg/dl, and the standard of hyperlipidemia is total cholesterol > 200 mg/dl. However, there were only 28.6% patients were diagnosed as hyperlipidemia, which seemed a little ridiculous. I thought authors should provide the number of how many patients were treated with lipid lowering drugs.

A. This observation is appropriate, there was a mistake in data transcription. We apologize for this and we corrected the hyperlipidemia prevalence. Patients treated with lipid lowering drugs were 28.6% .

8. In discussion, authors should discuss the relationship of age, gender and carotid IMT, since these variables significantly influenced IMT.

A. We think these are well known and expected results, therefore we think they don’t need further comments.

9. Traditionally, it is thought that carotid atherosclerosis is an inflammatory response, and authors should discuss the relationship of inflammation and serum calcium.

A. We agree and we added a comment on this topic.
